# A comprehensive platform for quality control of botanical drugs (PhytomicsQC): a case study of *Huangqin Tang *(HQT) and PHY906

**DOI:** 10.1186/1749-8546-5-30

**Published:** 2010-08-20

**Authors:** Robert Tilton, Anthony A Paiva, Jing-Qu Guan, Rajendra Marathe, Zaoli Jiang, Winfried van Eyndhoven, Jeffrey Bjoraker, Zachary Prusoff, Hailong Wang, Shwu-Huey Liu, Yung-Chi Cheng

**Affiliations:** 1 PhytoCeutica, Inc., 5 Science Park, New Haven, CT 06511, USA; 2 Department of Pharmacology, Yale University School Of Medicine, New Haven, CT 06510, USA

## Abstract

**Background:**

Establishing botanical extracts as globally-accepted polychemical medicines and a new paradigm for disease treatment, requires the development of high-level quality control metrics. Based on comprehensive chemical and biological fingerprints correlated with pharmacology, we propose a general approach called PhytomicsQC to botanical quality control.

**Methods:**

Incorporating the state-of-the-art analytical methodologies, PhytomicsQC was employed in this study and included the use of liquid chromatography/mass spectrometry (LC/MS) for chemical characterization and chemical fingerprinting, differential cellular gene expression for bioresponse fingerprinting and animal pharmacology for *in vivo *validation. A statistical pattern comparison method, Phytomics Similarity Index (PSI), based on intensities and intensity ratios, was used to determine the similarity of the chemical and bioresponse fingerprints among different manufactured batches.

**Results:**

Eighteen batch samples of Huangqin Tang (HQT) and its pharmaceutical grade version (PHY906) were analyzed using the PhytomicsQC platform analysis. Comparative analysis of the batch samples with a clinically tested standardized batch obtained values of PSI similarity between 0.67 and 0.99.

**Conclusion:**

With rigorous quality control using analytically sensitive and comprehensive chemical and biological fingerprinting, botanical formulations manufactured under standardized manufacturing protocols can produce highly consistent batches of products.

## Background

Quality control for herbal extracts containing tens to hundreds of characteristic phytochemicals pose a challenge for developing robust quality control metrics [1,2]. Variations in climatic conditions, geographic locations, methods of harvest, processing and extraction contribute to differences in the composition of the final product. Quality of herbal formulations was mainly assessed by highly skilled herbalists using sensory analyses including smell, taste and texture. More recently, these organoleptic methods have been augmented by histological identification [3], plant genetics [4,5] and increasingly sophisticated chemical analyses such as thin layer chromatography (TLC), gas chromatography (GC) [6], capillary electrophoresis [7] and liquid chromatography (LC) and detection methods such as UV/VIS absorption [8], Raman spectroscopy [9], infrared absorption [10], evaporative light scattering and mass spectrometry (MS) [11-14]. A typical certificate of analysis for an herbal formulation contains organoleptic information, TLC markers, specifications for water content, water and alcohol soluble extractives, total and acid soluble ash content, heavy metal analysis, microbial test, pesticide analysis and marker compound analysis as illustrated in a batch of PHY906 (Table 1). While these data are useful and generally accepted for herbal dietary supplements, they do not fully characterize the phytochemical composition or the biological response of the herbal extract.

**Table 1:** Certificate of Analysis

Test item	Specification	Result
General description	The product is a brown-colored powder possessing a little sweet taste	Passed
Identification	Identify Rf value and absorb spots of TLC to reference standards	Passed
Loss on drying	Not more than 10.0%	Passed
Water-soluble extractive	Not less than 60.0%	Passed
Dilute alcohol-soluble extractive	Not less than 60.0%	Passed
Total ash	Not more than 8.0%	Passed
Acid-insoluble ash	Not more than 2.0%	Passed
**Limit tests**		
Heavy metals (total)	Not more than 50 ppm	Passed
Copper (Cu)	Not more than 50 ppm	Passed
Arsenic (As)	Not more than 5 ppm	Passed
Cadmium (Cd)	Not more than 2 ppm	Passed
Mercury (Hg)	Not more than 0.5 ppm	Passed
Lead (Pb)	Not more than 20 ppm	Passed
**Microbial tests**		
A. Bacteria count (colonies/g)	A. Not more than 10000/g	Passed
B. *Samonella *species and *Escherichia. coli*	B. Negative	Negative
Identification	1) Identify HLPC chromatogram retention time match to reference standards	Passed
	2) Marker 1 > 50.0 mg/g	Passed
	Marker 2 > 7.0 mg/g	Passed
	Marker 3 >5.3 mg/g	Passed
Pesticide residues	Total BHC's: Not more than 0.2 ppm	Not detected
	Total DDT's: Not more than 0.2 ppm	Not detected
	PCNB: Not more than 0.2 ppm	Not detected

A typical Certificate of Analysis was supplied by the manufacturer of PHY906. Although these conventional tests provide specifications for botanical identification, general extraction information, specific heavy metals, microbial contamination, pesticide contamination and specific marker compounds, it does not provide a comprehensive chemical and biological profile of the extract for the purposes of quality control.

While the current standards for quality controls utilizes absolute quantitation of a few specific chemical marker compounds [14], there is increasing interest in using complete fingerprint patterns to characterize more completely the multi-chemical species [15]. However, no single analytical chemical method has high enough sensitivity and resolution to detect every potential phytochemical class of molecules.. Thus, an orthogonal biological methodology would be a useful complementary QC metric requirement. A robust bioresponse fingerprint incorporating living cells as the biological 'detector' and the resulting genomic differential display profile [16,17] after exposure to the botanical extract could provide a sensitive and global biological metric that may help validate batch-to-batch similarity and establish quality standards.

**Figure 1:**
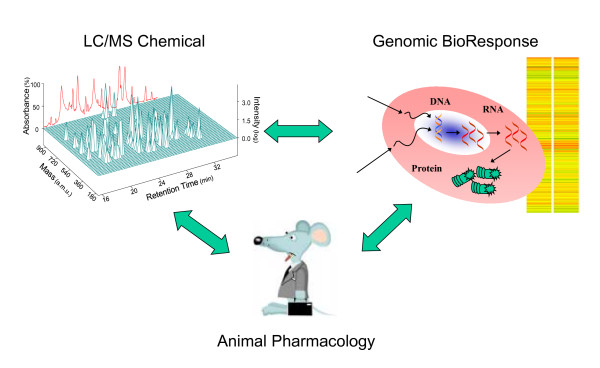
**PhytomicsQC**. PhytomicsQC integrates technologies for chemical marker compound analysis and chemical fingerprints, comprehensive bioresponse fingerprints and *in vivo *animal pharmacology validation. Currently, it combines LC/MS analysis to provide a global phytochemical fingerprint and a bioresponse differential gene expression profile to establish a multiplexed, quantitative metric for botanical quality control. A relevant animal model is used to define and validate the quality control metric and to help set batch acceptance criteria. Information-rich patterns are analyzed and compared with an established, well-characterized batch used for clinical studies. A statistical similarity score based on the ratios of the various measured data values within the pattern and varying typically between 0.0 and 1.0 is used to define pass/no-pass criteria for both the chemical and biological fingerprints.

PhytomicsQC is a methodology combining chemical analysis, bioresponse analysis and animal pharmacology to determine batch-to-batch reproducibility (Figure 1). Thus, it is a unified platform integrating: (1) information-rich chemical and bioresponse fingerprints, (2) molecular resolution details, (3) robust technologies (4) quantitative data, and (5) statistical pattern comparisons. For chemical analysis and fingerprinting, LC/MS was chosen for its sensitivity, broad capability and spectral sensitivity. Differential gene expression was selected for bioresponse fingerprinting (PCT US99/24851) for its comprehensive response, biological sensitivity and standardized methodology.

*Huangqin Tang *(HQT) is a classical Chinese medicine formula for treating gastrointestinal ailments including diarrhea, nausea and abdominal cramps [18]. PHY906 is a modified pharmaceutical preparation of HQT (US Patent No. 7,025,993). PHY906 reduces gastrointestinal toxicity and enhances the anti-tumor efficacy of some anti-cancer drugs in animal models [19-21] and is currently under clinical investigations [22-24].

The present study aims to describe and exemplify the PhytomicsQC approach to the quality control of herbal formulae using the example of HQT and its pharmaceutical derivative PHY906.

## Methods

### Herbal materials

A total of 18 batches of HQT were included in the present study. Four batches coded as PHY906-6, 7, 8, 10 were manufactured with PhytoCeutica's proprietary SOP. Eight batches of HQT were purchased from Sun Ten Pharmaceutical Co. LTD in Taiwan and designated as HQT-E, F, G, H, I, J, K and L. Six batches of HQT were obtained from various vendors (Chung Song Zong, Ko Da, Min Tong, Sheng Chang, Sheng Foong, Kaiser; Taiwan) who did not provide quality information, and were labeled as HQT-CSZ, KD, MT, SC, SF and KP3. The proprietary standard operating procedures (SOP) by PhytoCeutica for PHY906 used hot water extraction (80°C) of four herbs, namely *Scutellaria baicalensis *Georgi (S), *Paeonia lactiflora Pall. *(P), *Glycyrrhiza uralensis Fisch. *(G) and *Ziziphus jujuba Mill. *(Z) (ratio 3:2:2:2). The hot water extraction is then spray dried with insoluble dextran into a granulated powder, packaged and stored in foil containers at 4°C.

Chemical standards including baicalin (S), baicalein (S), wogonin (S), scutellarin (S), glycyrrhizin (G), ononin (G), liquiritin (G), liqiritigenin (G), paeoniflorin (P) and albiflorin (P), were obtained from Chromadex (USA). Apigenin and formic acid were obtained from Sigma-Aldrich (USA). Solvents were of LC/MS grade from JT Baker (USA).

### Extraction

Dried PHY906 or HQT powder (100 mg) was dissolved in one mL of 80°C water. The mixture was vortexed for one minute, placed in an 80°C water bath for 30 additional minutes with one minute of vortexing for every ten minutes. The sample was then cooled in a water bath of ambient temperature for five minutes, centrifuged for ten minutes at 10,000 rpm (Eppendorf Model 5810R, USA) and the resulting supernatant was filter (0.2 μm) sterilized. For subsequent LC/MS analysis, a 20 μL aliquot of this light brown extract was diluted with 980 μL of water. The final nominal concentration after extraction and dilution was 2 mg of dry weight PHY906 or HQT powder extract per mL of water. For biological experiments, the 100 mg/mL nominal concentration solution stock was diluted in the appropriate buffer or medium to the required final concentration.

### LC/MS methodology

High-performance liquid chromatography (HPLC) was performed with a Waters (USA) CapLC XE Pump equipped with a CapLC autosampler and a Waters (USA) CapLC 2996 Photodiode Array Detector. The eluents were (A) 100% water with 0.1% formic acid and (B) 100% acetonitrile with 0.1% formic acid and the column was a Waters Atlantis dC18 3 μm 0.3 mm × 150 mm NanoEase column (USA). The column was heated to 40°C and was preceded by a 0.5 μm precolumn frit. Gradient elution from 0 to 50% B over 70 minutes at 8 μL/min was used with an initial hold of five minutes. The column was then ramped to 95% B over four minutes, held in place for two minutes and returned to initial conditions over two minutes. Total run time was 120 minutes. Electrospray ionization was performed on a Micromass (UK) Q-Tof-II mass spectrometer. Samples (0.5 μL) were introduced without splitting into the electrospray interface through a 60 μm stainless steel capillary tube. A positive capillary voltage of 3.25 kV was used in positive ion mode and a negative capillary voltage of 3.25 kV was used in negative ion mode. The electrospray source was heated to 80°C and the desolvation gas (N_2_) was heated to 150°C at a flow rate of 400 L/hr. The Q-Tof was scanned from 50-2000 amu over one second. The resolution of the instrument under these conditions was ~10,000. For exact mass measurements, a reserpine lock mass ([M+H] of 609 amu) was introduced at the electrospray interface allowing mass measurements to be within 0.0002 amu. With external standards, mass accuracy to 0.002 amu was routine with experimental and theoretical mass matching accuracy of 20 ppm or better.

### Cell culture for gene expression studies

Three cell lines, namely Jurkat (ATCC no TIB-152), KB (ATCC no CCL-17) and HepG2 (ATCC no HB-8065), were selected for the experiments. HepG2 was selected for three reasons: (1) the cell line is stable, robust and well characterized; (2) the number of differentially expressed genes in HepG2 is generally observed to be higher than in the other two cell lines and (3) the liver is considered the primary drug-metabolizing organ for oral drugs. The HepG2 hepatocellular carcinoma cell line was cloned and a cell-bank created. A strict set of SOPs were developed to ensure reproducible growth characteristics including passage number and cell density. A HepG2 sub-clone cell was thawed with three passages to 80% confluency in 10% FBS complete MEME media at 37°C with 5% CO_2_. Computed IC_50 _values (concentration required to inhibit cell growth by 50%) were based on three independent experiments comparing a 72-hour exposure of the cells to eight concentrations ranging from 0.001 to 10 mg/mL of the PHY906-6 extract with control untreated cells. Cells were stained with 0.5% methylene blue, lysed with 1% sarcosine and cell viability determined by UV/VIS absorbance at A_595_.

### GeneChip experiments

Three independent experiments were performed on the HepG2 cells treated with one IC_50 _dose of the herbal extract or control buffer for 24 hours. At this time point, 100% of the cells were still viable. RNA was collected for gene profiling. GeneChip hybridization experiments with Affymetrix Human genome chip U133A (USA) were carried out at the Affymetrix Resource Laboratory, Yale University School of Medicine, USA. Data were processed with Microarray Suite 5.0 (Affymetrix, USA) software to generate a list of candidate genes for further investigation.

### Quantitative real-time polymerase chain reaction (qRT-PCR) experiments

Selected gene probes were purchased as Assays-on-Demand from Applied Biosystems (USA) to confirm and quantify the candidate genes identified in the GeneChip experiments.

### Animal studies

PHY906-6, 7, and 8 and HQT-F were compared for their effectiveness in potentiating the antitumor activity of the cancer chemotherapy drug CPT-11 or Camptosar^® ^(Pfizer, USA). Female BDF-1 mice (Charles River Laboratories, USA) of 4-6 weeks old (16-20 grams) implanted with murine Colon 38 colorectal cancer cells (National Cancer Institute, USA) were used in the experiments. Colon 38 cells were grown in RPMI 1640 medium (JRH Biosciences, USA) supplemented with 10% fetal bovine serum and 100 μg/ml kanamycin. Cells were maintained at 37°C in a humidified atmosphere of 5% CO_2_:95% air. For studies of the effects of PHY906 on antitumor efficacy and toxicity, Colon 38 cells (1-2 × 10^6 ^cells in 0.1 ml phosphate-buffered saline, PBS) were transplanted subcutaneously (sc) into the BDF-1 mice. The length and width of the tumors were measured with a sliding caliper. The tumor size (S) was estimated according to the formula as follows:

$$\text{S}=\text{L}\times {\text{W}}^{\text{2}}\text{/2}$$

where L is length, W is width.

After 10 to 14 days, mice with tumor sizes of 150-300 mm^3 ^were selected. Treatment groups consisted of five mice each. Tumor size, body weight and mortality of the mice were monitored daily. Mice were sacrificed when the tumor size reached 10% of the body weight.

PHY906 was administered *per oral *(po) whereas Camptosar^® ^was administered intraperitoneally (ip). PHY906 was given twice daily (bid) at approximately 10 am and 3 pm. On days when Camptosar^® ^was also administered, PHY906 was given 30 minutes earlier. Unless otherwise indicated, dosages were 500 mg/kg for PHY906 and 360 mg/kg for Camptosar^®^. Mice in the control groups were administered a vehicle of either PBS (ip) or water (po). All animal studies were conducted at the Yale University Animal Facility and approved by the Institutional Animal Care and Use Committee.

### Pattern comparison by R value and Phytomics Similarity Index (PSI)

The linear correlation R value is a standard statistical method [25] used to compare two datasets and to compare the absolute intensity or value of each of the collected (N) data points. These data points can be either ion current spectral intensities collected by LC/MS, UV-VIS or relative gene expression level values determined by qRT-PCR. The R value varies between -1.0 (perfect anti-correlation) and 1.0 (perfect correlation) and is a measure of the similarity of the two sets of intensities. The Phytomics Similarity Index (PSI) is also a statistical method that compares the fingerprint patterns by computing a correlation value not of the intensities of the N peaks but rather on the ratio data computed for each of the N data points with each of the other (N-1) data points. Using these (N-1) ratio values in the computation for each of the N data points provides the similarity of that peak in relation to all of the other peaks in the fingerprint pattern (PCT US02/34121) The ratio information is incorporated into the analysis as it provides relative information between various peak intensities reflecting the importance of the balance of the compound amounts (or gene expression levels). As an example, the integrated ion counts for each of the N peaks (mass and retention time) are extracted from the overall spectra of two different batches (A and B). These N ion intensities, representing the chemical fingerprint of each batch, are placed, conceptually, along the diagonal of a matrix of dimension N × N and the ratios of the intensities are placed in the assigned M_i,j _(i ≠ j and i, j ≤ N) off-diagonal matrix locations. There are, therefore, a total of N(N-1)/2 unique non-diagonal elements describing the full set of intensity ratio information between all of the peaks with each peak contributing (n-1) ratios. Matrices A and B were respectively designated as M^A ^and M^B^. Each column/row in M^A ^and M^B ^may be represented by the vectors as follows:

$$\begin{array}{l} x_{i}^{A}=(M_{i1}^{A},M_{i2}^{A},M_{i3}^{A},M_{i4}^{A},M_{i5}^{A},\cdots M_{ij}^{A},\cdots M_{iJ}^{A}|i\neq j)\\ x_{i}^{B}=(M_{i1}^{B},M_{i2}^{B},M_{i3}^{B},M_{i4}^{B},M_{i5}^{B},\cdots M_{ij}^{B},\cdots M_{iJ}^{B}|i\neq j) \end{array}$$

The linear correlation is then computed using all of the columns or rows in both matrices.

$$R=\frac{n\sum x_{A}x_{B}-\sum x_{A}\sum x_{B}}{\sqrt{\left(n\sum x_{A}^{2}-{\left(\sum x_{A}\right)}^{2}\right)\left(n\sum x_{B}^{2}-{\left(\sum x_{B}\right)}^{2}\right)}}$$

The correlation value R for each column i.e. peak, can be obtained with the standard Pearson coefficient or the Spearman ranked coefficient [25]. The result of this analysis is a vector of R scores, where each vector element corresponds to a data point (e.g. MS peak, or gene) that is common to both datasets. While each data point (i) has its own correlation score, R_i_, the average of all of the individual R scores produces a diagnostic single value for similarity defined as the PSI. In this example, the PSI score would range between 0.0 (complete dissimilarity) to 1.0 (complete identity) to -1.0 (perfect anti-correlation). The individual PSI values can be weighted by a variety of factors including intensity, slope or biological importance. A weighting function found to be valuable is the individual peak slope calculated from plotting (n-1) ratios for peak i batch A to the equivalent (n-1) ratios for peak i in batch B. Highly similar batches tend to have PSI values greater than 0.85 with only a few outliers at lower PSI values. Batches that have poor similarity tend to have PSI values less than 0.75 with a greater number of individual outliers at lower PSI values. The PSI algorithm along with tools for filtering and sorting the LC/MS data were implemented in the software package PhytomicsQC™.

## Results

### PHY906 extraction

**Figure 2:**
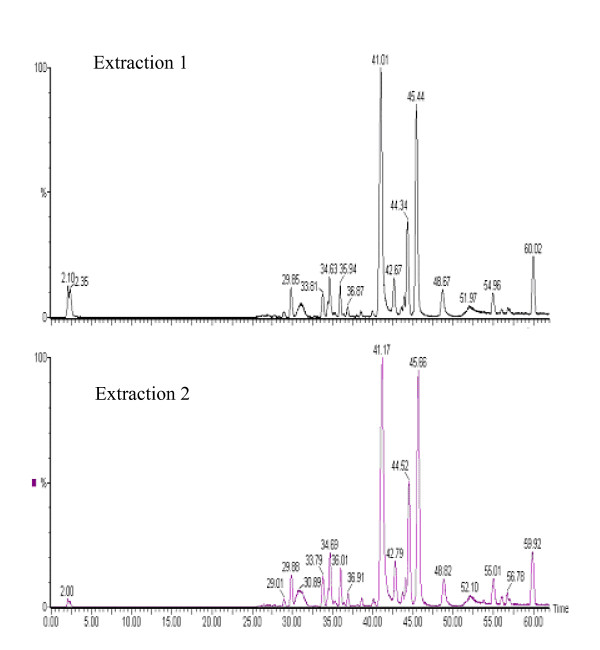
**LC/MS Chromatograms of Multiple Extractions of PHY906**. Extraction efficiency of PHY906 spray-dried extract. PHY906-6 powder was extracted with 80°C water (100 mg/ml) for 30 minutes. The remaining solid after a high speed spin of 10,000 rpm was extracted a second time with 80°C water for 30 minutes. LC/MS(+) spectra of each liquid extract indicate very similar peak patterns The efficiency for each extraction was approximately 80% as determined by dilution factors to maintain the TIC at 1.7e4 (1:50 for the first extraction and 1:5 for the second extraction) and by recovered masses.

Multiple extractions of PHY906 exhibited similar LC/MS profiles and indicated an extraction efficiency of 85% with a composition greater than 80% low molecular weight (<1000 amu) phytochemical species. (Figure 2) The high extraction efficiency and the similarity of the phytochemical profiles from multiple extractions suggested that the soluble sample was an excellent representation of the phytochemical components.

### Phytochemical analysis

**Figure 3:**
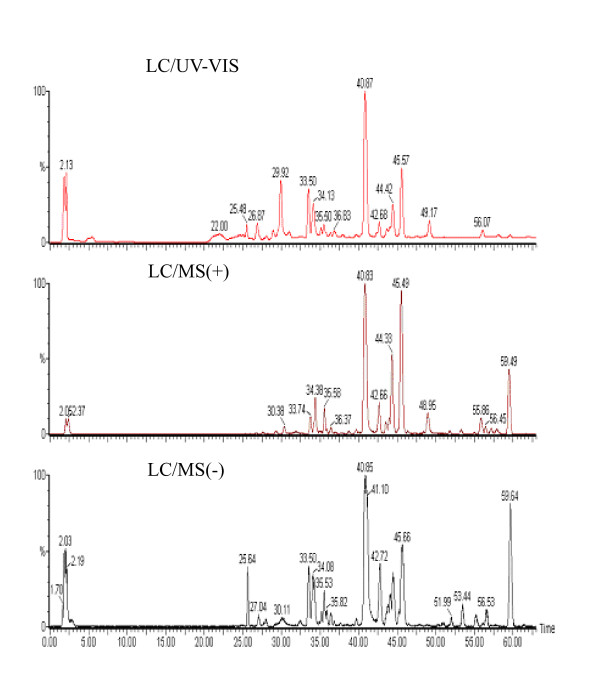
**MS and UV/VIS Detection of PHY906**. Three detection modes were employed to detect the spectrum of phytochemicals in PHY906 extracts. The top panel illustrates detection in the UV/VIS range using a photo diode array detector (200-400 nm). The middle panel illustrates detection by MS(+) with a TIC of 1.5e4. The lower panel illustrates detection by MS(-) with a TIC of 2.5e3. UV/VIS detection was poor for many of the saponins and triterpenoids associated with (G) and was unable to detect or resolve the marker for (Z) in the solvent front. Only 22 of the 39 peaks in the final chemical fingerprint were detected and no new peaks were observed. MS(+) detection was approximately eight fold more sensitive than MS(-) by TIC resulting in increased S/N. 32 of the 39 chemical fingerprint peaks were observed in the MS(-) mode compared with the MS(+) mode. No new peaks were observed in the MS(-) mode although the intensity profile was enhanced for a few species including paeoniflorin sulfonate at 25.6 minutes.

Comparison of LC/UV-VIS spectra and positive (+) and negative (-) ion mode LC/MS spectra of PHY906 (Figure 3) indicated the presence of a similar pattern of peaks with various intensity profiles. LC-MS (+) detected 39 distinct and quantifiable peaks suitable for use in a chemical fingerprint. In contrast, LC-MS(-) revealed 32 of the 39 peaks found in positive-ion mode and no additional new peaks whereas UV/VIS detection revealed only 22 of the 39 peaks directly and no additional peaks.

### Sample stability

**Figure 4:**
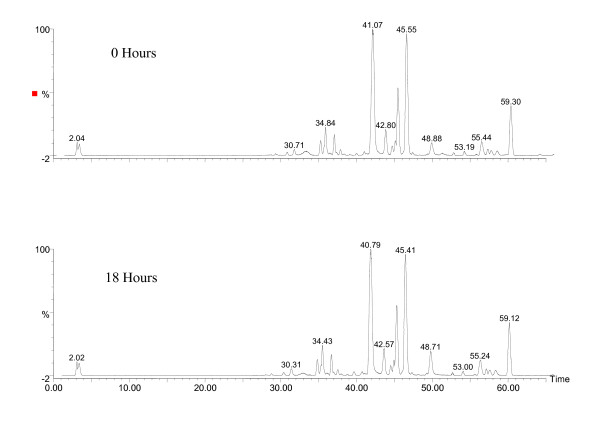
**LC/MS Chromatograms of PHY906 Extract Stability**. Sample stability. Sample and instrument stability were monitored by successive LC/MS(+) profiles of a freshly prepared extract of PHY906-6. Two spectra taken at 0 hours and 18 hours indicate that LC peak positions and peak integrations were stable, samples were visually unchanged with no observed precipitation and peak patterns and intensities did not vary over at least an 18-hour period. The PSI value for the 39 peak pattern between the 0 and 18 hour time points was 0.98. Even minor degradation of the liquid extract was not apparent for at least 24 hours at room temperature.

A freshly prepared extract of PHY906 was analyzed by LC/MS (+) and indicated no significant changes over a period of at least 18 hours (Figure 4). Samples stored at -80°C were stable for a period of at least one month at a concentration of 100 mg/mL.

### Chemical fingerprints

**Figure 5:**
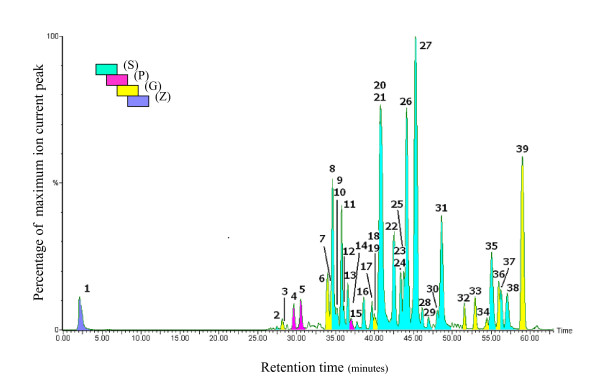
**LC/MS chromatogram of PHY906-6**. LC/MS(+) spectrum of PHY906 extract and herbal source identification. Thirty-six peaks were resolved and 64 compounds were identified or tentatively identified (23). Thirty-one peaks were found to contain a single molecular species while 5 peaks contain multiple molecular species. 39 compound peaks defined the chemical fingerprint and were used for batch-to-batch comparisons. Of the 39 peaks of the chemical fingerprint of PHY906 (S) accounted for 25 of 39 peaks, (P) accounted for 3 of 39 peaks, (G) accounted for 10 of 39 peaks and (Z) accounted for only 1 of 39 peaks. All the identified peaks had a unique retention time and/or mass signature and were associated with a single herbal ingredient. Water extracted (Z) was nearly devoid of resolved phytochemical peaks that could be detected. The single identified peak for (Z) was very hydrophilic, had no UV chromophore, eluted in the solvent front of the C18 reverse phase column and ionized only in (+) positive MS mode. The total ion count for the spectrum was 2.9e4. The complete chemical fingerprint of 39 peaks accounted for more than 82% of the total ions above a threshold of 1.5% of the largest peak.

A total of 64 LC/MS peaks were detectable in PHY906-6 [26] under current LC/MS conditions. A diagnostic chemical fingerprint pattern of 39 of the LC/MS (+) peaks was chosen for quality control. The peaks selected for the chemical fingerprint all had peak intensities greater than 0.2%, reproducible peak integration in three independent spectra and linearity over a ten-fold concentration range. Each of the 39 peaks identified in the PHY906 LC/MS (+) spectrum was unique to an individual herbal component; 25 from (S), 3 from (P), 10 from (G) and 1 from (Z) (Figure 5). These 39 peaks represented 77% of the total ion count (TIC), summed over the overall chromatogram from 0 to 65 minutes, at a threshold of 1%, 82% of the TIC at a threshold of 1.5% and 87% of the TIC at a threshold of 2.0%. A list of these 39 phytochemical peaks is in Additional file 1.

### Marker standards

Quantitative analysis was performed for six markers from (S), two markers from (G) and two markers from (P). No relevant marker from (Z) was available although one definitive marker peak is identified with mass 159.085 amu. Recovery studies reported a range between 96% and 105%. Standard curves for all markers were linear in the range 0.1 to 20 mg/ml with linear correlation R-values greater than 0.99. The ten marker standards accounted for approximately 20% of the total mass of PHY906, 38% of the total mass of phytochemicals after correction for excipient and residual water content and 58% of the total mass of phytochemicals excluding excipient, residual water content and simple sugars (See Additional file 1).

### Compound identification

Ten of the 39 peaks were identified and confirmed with external marker standards, high-resolution MS and MS/MS fragmentation. An additional 13 of 39 peaks were tentatively identified with high-resolution MS and/or MS/MS. These 23 peaks comprised 78% of the ion current intensity by all 39 peaks. The majority of these identified compounds were flavonoids (60%), saponins and triterpenoids.

### Bioresponse analysis

**Figure 6:**
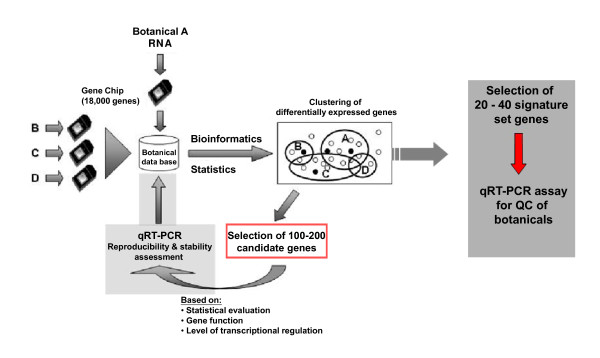
**Schematic for gene expression bioresponse fingerprint**. A Scheme of generating the bioresponse gene expression pattern for a botanical extract. The bioresponse of a living cell, provides a unique biological fingerprint of complex actions by the full extract of the botanical drug. The bioresponse can be one of many multifactorial responses, including differential gene expression, differential protein expression or post-translational modifications such as phosphorylation. We illustrate the process using living cells as "detectors" and genomic expression levels as the observed bioresponse. Well characterized gene chips (Affymetric UA133A) serve as the first filter to reduce the 18,000+ possible genes down to the candidate gene expression pattern of 100-300 genes. This gene list is then compared against a botanical bioresponse database, filtered and analyzed to produce unique sets of bioresponse genes. This list is further refined by statistical evaluation, gene function, transcriptional level, relevance, etc. before validation with qRT-PCR. This iterative process generates a signature set of 15-30 genes that are stable, quantitative, reproducible and unique to both the botanical formulation and manufacturing process.

Of the approximately 18,000 genes monitored, only 100-300 genes were significantly regulated as indicated by an over 1.5 fold change in the differential gene expression level in HepG2 cell culture in the presence of a one IC_50 _dose of an herbal extract over a period of 24 hours. This list of genes was further filtered by reproducible qRT-PCR and comparative gene function analysis to form a unique signature set of 15-20 genes (Figure 6).

### Gene expression

**Figure 7:**
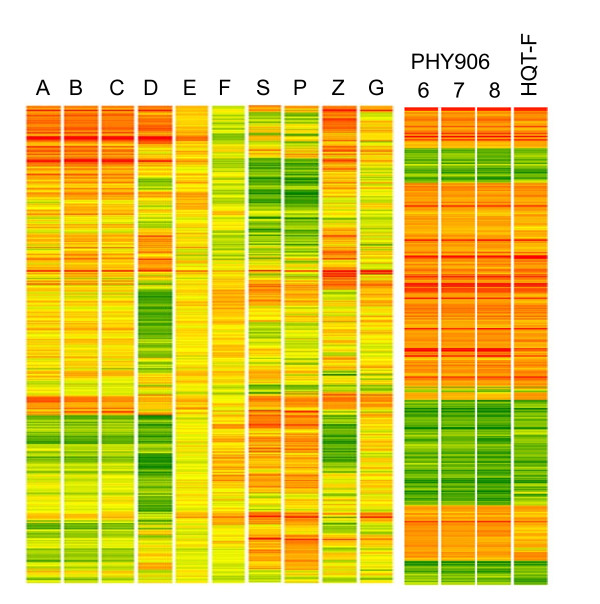
**Gene expression bioresponse profiles**. Composite union gene expression of ten different herbal preparations. Ten different herbal preparations including three forms of Ginseng (A) White, (B) Red, (C) American, (D) Cistanche *tubulosa *(Schenk) R. Wight, (E) *sinensis sinensis*, (F) *Ganoderma Lucidium*, (S) *Scutellaria baicalensis*, (P) *Georgi Paeonia lactiflora Pall*., (Z) *Ziziphus jujuba Mill.*, (G) *Glycyrrhiza uralensis Fisch.*, PHY906-6, 7, 8 and HQT-F were examined. Each preparation was used to treat HepG2 cell cultures for a period of 24 hours at the standard IC50 dose for the herbal or formulation with gene expression levels measured using the Affymetric UA133A chip. Combining data from eleven different herbs or herbal formulations generated a total of 524 genes in the union set that are regulated with greater than a 1.7 fold change compared with a buffer-treated control. This color-coded gene expression map shows the unique expression patterns for these 524 genes observed for different herbal preparations. While high similarity was observed for the three ginseng varieties, there were still subtle differences that distinguished the varieties. Similarly, although three clinical batches PHY906-6, 7 and 8 were nearly identical, there were subtle differences compared with the bioresponse gene expression pattern of HQT-F.

Gene response expression data observed at an exposure of one IC_50 _concentration of eight herbs resulted in a composite bioresponse gene set of 524 genes at a minimum cut-off of 1.7 fold change in expression level (Figure 7). Unique gene expression patterns are evident for each herb or herbal formulation. A biochemical pathway analysis of these 524 genes suggested that over 50% of the genes were either in signaling pathways or involved in cellular metabolism. This gene-list represented an objective biological quality control metric for an herbal extract.

In the specific case of PHY906-6, three independent experiments revealed 1172, 1846 and 1158 regulated genes in HepG2 cells, of which 466 genes were common in all three experiments. Subsequent filtering of regulated genes with changes of 1.5 fold, 2.0 or 3.0 folds with respect to untreated control resulted in a surprisingly small common gene set of 261, 77 or 28 genes respectively. The set of 77 genes was filtered to a subset of 17 genes, 15 of which were confirmed by qRT-PCR analysis. Nearly all (14/15) of the altered genes were up-regulated. The full expression range for these 15 genes varied from 3-fold down-regulated to over-400-fold up-regulated (Table 2). The subset of 15 genes formed a unique bioresponse signature of the PHY906 extract as a quality control metric for quantitative batch-to-batch comparisons.

**Table 2:** PHY906 gene expression bioresponse in HepG2 cell-line

Protein name	Gene name	Cellular function	Fold change
Aldo-keto reductase family 1 member B10	AKR1B10	Metabolism	6.8
Carnitine palmitoyltransferase 1A	CYP1A1	Metabolism	405
Epithelial membrane protein 2	EMP2	Cell growth regulation	3.2
Glucose-6-phosphatase catalytic subunit	G6PC	Metabolism	12.3
Glutamate-cystein ligase catalytic subunit	GCLC	Metabolism	3.4
Growth differentiation factor 15	GDF15	Cell growth/differentiation	2.2
Hepcidin antimicrobial peptide	HAMP	Homeostasis, metabolism	4.9
Insulin-like growth factor binding protein 3	IGFBP-3-2	Hormone, Immune response,	3.3
Palladin	Palladin	Cell growth regulation	2.6
Serine/threonine protein kinase PIM1	PIM1	Signalling transduction and cell proliferation, oncogene	3
Sterile alpha motifs- and SH3 domain-containing protein 1	SASH1	Cell growth regulation	2.8
SERTA domain	SERTAD	transcriptional regulator	2.2
Solute carrier family 7 member 11	SLC7A11	Membrane transport protein	3.2
Son of sevenless homolog 1	SOS 1	Signalling transduction and cell death regulation	9.4
Tubulin, alpha 3	TUBA3	Signalling transduction and cell death regulation	-2.4

PHY906-6, at the IC_50 _dose (0.85 g/ml dry weight), or control buffer was applied to a standardized cell culture of HepG2 cells for 24 hr. No cell death was observed by methylene blue staining. Cells were harvested and RNA was isolated from both PHY906-6 treated and control treated cells. The RNA was quantitated using qRT-PCR and standardized gene probes from Applied Biosystems Assays-On-Demand for the 15 genes in the gene signature. Fourteen of the fifteen genes were up-regulated. The genes coded for proteins with a variety of cellular functions. No information regarding cellular mechanisms of action of PHY906 could be inferred from these data, as the data indicated the cellular bioactivity of the entire extract rather than the bioavailable fraction. The qRT-PCR data, however, were reproducible in an independent experiment within approximately 30%.

### Validation of the PSI method

The PSI method was tested and validated with artificial data sets created within the boundary conditions of typical experimental data. Two identical datasets produced a PSI value of 1.0. Random data sets provided low PSI values in the range of 0.0 to 0.1. Data values greater than ten provided a robust and stable score whereas five or fewer data points did not provide reliable results. PSI was accurate when the variations between the two datasets were spread over a majority of the data values. If only one of the data points was variable, both the PSI average and the R-value correlation were high. However, the data point was easily identified in the PSI histogram plot as a low value outlier.

### Batch-to-batch comparison-chemical fingerprints

The 39 peak chemical fingerprints were used to compare 17 batches of PHY906 and generic forms of HQT with a clinical standard batch PHY906-6. Mass spectra of all batches revealed subtle (but distinct) quantitative differences in the peak intensity pattern. With the extracted intensities for each of the 39 chemical fingerprints, we computed the PSI and conventional correlation values to compare similarity (Table 3). PSI values ranged from 0.67 to 0.99 whereas the correlation R values tightly clustered between 0.97 and 0.99. PSI values of 0.99 confirmed that the 39 peak chemical fingerprints of PHY906-6, 7 and 8 that were manufactured as sequential batches using the same ingredient herbs are nearly identical as chemical fingerprint patterns of two sequential LC/MS data sets of the same sample would have a PSI of 0.99. PHY906-10 was also found to be highly similar (PSI 0.97) to PHY906-6 although it was manufactured with herbs harvested six years later (group I). Similarly, the seven batches of group II manufactured by the same vendor as group I were also highly consistent with each other (PSI 0.95-0.98) but differed from the clinical batch PHY906-6 (PSI 0.0.81-0.94). The greatest variation, however, was between PHY906-6 and the six batches in group III (PSI 0.67-0.96) sourced from various vendors. Some batches such as HQT-SC, HQT-KP3 and HQT-KD were very similar to PHY906-6 with PSI scores greater than 0.90 while other batches such as HQT-MT and HQT-SF were significantly different with PSI scores less than 0.75. Without vendor information for these samples, it was impossible to determine product batch-to-batch reproducibility.

**Table 3:** PSI and R-values for the Chemical Fingerprints of Seventeen Batches of PHY906 and HQT

Formulation	PSI	R
**Group I**		
PHY906-6	1	1
PHY906-7	0.99	0.99
PHY906-8	0.99	0.99
PHY906-10	0.97	0.99
**Group II**		
HQT-E	0.94	0.99
HQT-F	0.81	0.98
HQT-G	0.84	0.97
HQT-H	0.87	0.98
HQT-I	0.89	0.98
HQT-J	0.84	0.98
HQT-K	0.82	0.98
HQT-L	0.86	0.98
**Group III**		
HQT-CSZ2	0.89	0.99
HQT-SF	0.67	0.97
HQT-SC	0.95	0.99
HQT-MT	0.74	0.98
HQT-KP3	0.93	0.99
HQT-KD	0.96	0.99

The chemical fingerprint intensity pattern of 39 peaks is used to compare the similarity of seventeen independently manufactured batches of PHY906 or HQT with the clinical batch PHY906-6. Group I consists of four clinical batches: PHY906-6, 7, 8 and 10. Group II consists of eight batches manufactured for sale as Huang Qin Tang by a single vendor: HQT-E, F, G, H, I, J, K and L. Group III consists of six batches that are reported to be Huang Qin Tang and that are manufactured by different vendors with unknown protocols, specifications or quality control: HQT-CSZ, KD, MT, SC, SF and KP3. A PSI value of 1.0 indicates identical patterns of the intensity ratio pattern of the chemical fingerprint between the PHY906-6 and a second batch. A PSI value of 0.0 indicates no similarity of the intensity ratio pattern between the two batches. Batches in Group I are found to be highly similar to PHY906-6 including PHY906-10 that is manufactured six years after batches PHY906-6, 7, 8 using different harvests of the raw herbal starting products. Group II are relatively tightly clustered at a lower PSI value, and while similar to each other are clearly distinguishable from Group I. Group III are poorly clustered, highly variable and span the largest PSI range (0.67 - 0.95). While some batches are very similar to PHY906-6, other batches are quite different. In the lower panel is a full matrix of PSI values comparing PHY906-6, 7, 8, 10 and HQT-F. Intra-batch comparisons indicate the high degree of similarity of the clinical batches (Group I) and the lower degree of similarity of Group I batches with HQT-F.

### Comparison of PSI and R value

Although a very modest correlation (R^2 ^= 0.81) existed between PSI values and R values, the small range of R values could not be used definitively to discriminate between various batches of HQT. The PSI was apparently more sensitive to variations in the intensity pattern because each of the n peaks had (n-1) ratios used in defining the correlation coefficient with the corresponding peak in a separate batch while in the standard R value each peak intensity only contributed 1/n to the overall correlation coefficient.

### Batch-to-batch comparison-bioresponse fingerprints

Three clinical batches PHY906-6, 7 and 8 and two non-clinical batches HQT-E and F were selected for bioresponse fingerprint analysis as they were all manufactured by a single vendor with batch HQT-E exhibiting the highest chemical fingerprint similarity (PSI = 0.94) and HQT-F the lowest chemical fingerprint similarity (PSI = 0.81) compared with PHY906-6. Bioresponse PSI values computed with qRT-PCR data of the 15 gene expression pattern were 0.99 for PHY906-7, 0.98 for PHY906-8, 0.94 for HQT-E and 0.68 for HQT-F compared with PHY906-6 (Table 4). This complementary bioresponse fingerprints confirmed the rank-order similarity observed in the chemical fingerprints.

**Table 4:** PSI of 15 Gene Expression Bioresponse Fingerprint of PHY906 and HQT batches

	PHY906-6	PHY906-7	PHY906-8	HQT-E	HQT-F
PHY906-6	1	0.99	0.98	0.94	0.68
PHY906-7		1	0.97	0.92	0.71
PHY906-8			1	0.97	0.61
HQT-E				1	0.58
HQT-F					1

Five batches were selected from the total of 17 batches, based on chemical fingerprint similarity. Three batches from Group I (PHY906-6, PHY906-7, PHY906-8) that exhibit high similarity were chosen. Two batches, HQT-E and HQT-F from Group II were selected that were manufactured by the same vendor. HQT-E exhibited the highest similarity and HQT-F exhibited the lowest chemical fingerprint similarity to PHY906-6. Differential gene expression values for the 15 gene bioresponse fingerprint were measured by qRT-PCR using Assay-on-Demand from Applied Biosystems, standardized HepG2 cells and a one IC50 dose level of the PHY906 or the HQT batch. PSI values are based on the Pearson correlation between the ratio matrices of the 15 differential gene expression changes and vary between 0.0 (no similarity) and 1.0 (complete identity). The gene expression values ranged between -2.4 and 405 and are shown in supplemental table 3 for PHY906-6. Duplicate experiments with either PHY906-6 or HQT-F results in PSI values of 0.97 indicating a high level of reproducibility. Comparison of PSI values using this 15 gene fingerprint indicate high similarity (0.92-0.99) between batches PHY906-6, PHY906-7, PHY906-8 and HQT-E, but significantly lower similarity (0.58-0.70) with batch HQT-F.

### Batch-to-batch comparison

**Figure 8:**
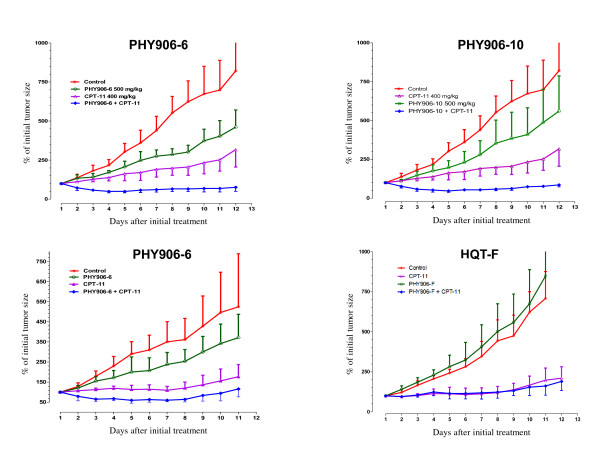
**Tumor growth in BDF-1 mice**. Effects of three herbal batches on Camptosar^® ^anti-tumor activity in mice. PHY906-6, PHY906-10 and HQT-F were tested to monitor enhancement of the activity of Camptosar^® ^on solid colon-38 tumors *in vivo*. BDF-1 mice (20-22 grams) with tumor sizes of 150-300 mm^3 ^were selected. Five mice were used in each of four groups: (1) control (PBS for intraperitoneal or water for oral), (2) PHY906 or HQT only (500 mg/kg), (3) Camptosar^® ^(360 mg/kg) and (4) Camptosar^® ^(360 mg/kg) and PHY906/HQT (500 mg/kg). PHY906 was given orally (po) whereas Camptosar^® ^was administered intraperitoneally (ip). Two different animal studies were conducted. The first study compared PHY906-6 with PHY906-10 (upper panel). The results of this study indicate that both PHY906-6 and PHY906-10 enhance the anti-tumor effect of Camptosar^® ^compared with Camptosar^® ^alone (*P *= 0.0001). A second independent study compared PHY906-6 with HQT-F (lower panel). The results indicate that PHY906-6 enhances the anti-tumor effect of Camptosar^® ^when compared with Camptosar^® ^alone (*P *= 0.0001). There was no significant enhancement by the HQT-F batch (p = 0.386)

Based on chemical and bioresponse analysis, three batches (PHY906-6, PHY906-10 and HQT-F) all produced by the same manufacturer, were used to investigate the effects on the anti-tumor activity of Camptosar^® ^against murine colorectal cancer in mice (Figure 8). There was a significant efficacy enhancement for Camptosar^® ^by batch PHY906-6 and batch PHY906-10 (P = 0.0001) but no significant enhancement by batch HQT-F (P = 0.386) as determined by the paired t-test. These *in vivo *results were consistent with the similarity ranking in both *in vitro *chemical and bioresponse fingerprints.

## Discussion

The challenge of assessing the consistency of different batches of a botanical extract is inherent in the phytochemical complexity of botanical extracts. This challenge is made more formidable due to the fact that two batches of a botanical extract with the same chemical spectrum may have different biological activities if the bioactive chemical species is not detectable by the specific chemical analysis methodologies. Similarly, two batches of a botanical extract with different chemical fingerprint compositions may exhibit the same biological activity if the phytochemicals responsible for the difference are biologically inert. This challenge demands comprehensive quality control of polychemical botanical extracts to include multiplexed and orthogonal methods for both chemical and biological characterization.

While the traditional chemical analysis of standard marker compounds provides a useful quantitative mass balance, patterns of information-rich chemical fingerprints provide a complementary, powerful and practical approach to herbal quality control. Well suited for the analysis of the phytochemical-rich extract of PHY906, LC-MS offers excellent sensitivity, molecular resolution and good reproducibility in providing a comprehensive chemical fingerprint pattern. Other information-rich analytical chemical methods such as LC-NMR, UV-VIS and FT-IR are also useful. However, while these methods are well suited for the characterization of low molecular weight, phytochemical-rich botanical extracts, these chemical analysis may not be well suited to fully characterize the complex and heterogeneous protein/carbohydrate profiles often associated with important herbal or fungal extracts. A complementary biological methodology is required.

Comprehensive biological methodologies such as a quantifiable and global bioresponse fingerprinting are more advantageous than a few specific single enzyme/receptor assays. The advantages are due primarily to the inherent multi-factorial biological activities of botanical extracts. Even in the absence of a complete understanding of the exact bioactive chemical species and the underlying mechanisms of action, the global fingerprints provide a comprehensive and objective assessment of an herbal extract according to quality control metrics. As illustrated by the example of PHY906, the results indicate that a sensitive cellular detector and a gene expression readout is a useful approach to characterizing an integrated bioresponse of macromolecule-rich extracts found in various fungal extracts. Examination of multiple cell types as potential "detectors" revealed that these complex polychemical mixtures only regulate a few hundreds of genes out of a total of ~18,000 possible genes. This list of a few hundred genes could be filtered down to a smaller subset of genes to form a selective, unique and quantifiable bioresponse signature pattern. Interestingly, we found no obvious similarity in the gene expression bioresponse pattern for any of the individual herbal ingredients used in the manufacture of PHY906 as compared with the complete PHY906 formulation. This finding suggests that the bioresponse of PHY906 mixture, is more complex than the simple summation of the individual bioresponses of the ingredients.

The ability to manufacture consistent batches of herbal extracts is fundamental to evidence-based scientific and clinical study of botanical extracts. The problems of botanical extract consistency [27-29] are mainly due to poor product manufacturing protocols or non-standard manufacturing procedures. The results of this study of eighteen different batches of HQT confirm that significant differences could be observed from samples from different vendors. However, the analysis  also strongly indicates that when careful sourcing of botanical ingredients and standardized manufacturing protocols are employed, that multiple batches of  a complex botanical formulation, produced in different years and with different harvests of raw herbal ingredients, can also be highly consistent. The present study suggests that herbal batches with chemical fingerprint PSI similarity scores greater than 0.85 are likely to be pharmacologically bioequivalent.

Chemical fingerprints and bioresponse fingerprints corroborated by an *in vivo* pharmacology model, provide orthogonal and complementary characterization methodologies for determining batch-to-batch similarity. Both LC/MS and qRT-PCR are standardized, highly reproducible and cost-effective for characterizing pharmaceutical botanical extracts. While neither methodology by itself is sufficient to characterize a botanical extract, the combination of chemical and biological characterization does provide information-rich, high resolution metrics for comparing different batches of an herbal extract.

PhytomicsQC will be continually improved. The next generation of the PhytomicsQC platform will include sophisticated data mining tools and multiplexed chemical and biological response fingerprints to identify the biologically active subset of the chemical fingerprints and utilize PSI values that combine chemical and biological information..

## Conclusion

PhytomicsQC is a first generation platform for botanical quality control that integrates high resolution, global chemical fingerprints, novel bioresponse genomic expression fingerprints, *in vivo *validation and a statistical pattern comparison algorithm, to provide an information-rich approach to determining the batch-to-batch similarity of botanical extracts. When this comprehensive methodology was used to analyze HQT and its pharmaceutical derivative PHY906, some significant differences were found between herbal batches from different manufacturers. However, when herbal selection and manufacturing are carefully controlled, batches manufactured years apart could be highly similar in their chemical, cellular response and pharmacological profiles.

## Abbreviations

S: *Scutellaria baicalensis *Georgi; P: *Paeonia lactiflora Pall*; G: *Glycyrrhiza uralensis Fisch*; Z: *Ziziphus jujuba Mill*; QC: Quality Control; HQT: Huangqin Tang; po: per oral or by mouth; ip: intraperitoneally; bid: "bis in die"; Latin for twice a day; PSI: Phytomics Similarity Index; UV-VIS: Ultraviolet-Visible; MS: Mass Spectrometry; LC/MS (+) (-): Liquid Chromatography coupled Mass Spectrometry (positive mode) (negative mode); TIC: Total Ion Current; HPLC: High Pressure Liquid Chromatography; GC:Gas Chromatography; TLC: Thin Layer Chromatography; IACUC: Institutional Animal Care and Use Committee; PSI: Phytomics Similarity Index

## Competing interests

The authors of this paper are associated with PhytoCeutica, Inc.; YCC is the scientific founder and the other authors are or were employees of PhytoCeutica, Inc. RT, SHL and YCC hold stock or stock options in the company.

## Authors' contributions

RT developed the PSI methodology. AP and JG conducted the LC/MS characterization of HQT and PHY906. RM and WE conducted the bioresponse gene expression fingerprints and quantitative PCR experiments. ZJ and SHL performed the animal pharmacology experiments. JB and HW developed the code and validated the PSI algorithm and implemented the PhytomicsQC platform software. ZP, AAP and RT conducted data analysis including PSI comparisons. YC developed the concept of phytomics and invented the bioresponse gene expression analysis. All authors read and approved the final version of the manuscript.

## Supplementary Material

Additional file 1 Chemical fingerprint of PHY906 Click here for file
