# Supplementary material for: A comprehensive platform for quality control of botanical drugs (PhytomicsQC): a case study of Huangqin Tang (HQT) and PHY906
Source: Chin Med. 2010 Aug 20;5:30. doi: 10.1186/1749-8546-5-30 (PMC2940884; doi:10.1186/1749-8546-5-30)
Supplement: Additional file 1 — Chemical fingerprint of PHY906 [file 1749-8546-5-30-S1.DOC]

**Additional file 1-** Chemical fingerprint of PHY906

| **ID** | **Time** | **Formula** | **Mass** | **Herbal source** | **Chemical name** | **Chemical class** | **Conc. mg/gr** | **Relative %** |
| --- | --- | --- | --- | --- | --- | --- | --- | --- |
| 1 | 2.3 | C7H13NO3 | 159.085 | Z |  |  |  | 4.30 |
| 2 | 26.57 | C15H12O7 | 304.048 | S |  |  |  | 0.64 |
| 3 | 26.57 | C21H20O10 | 432.118 | G |  |  |  | 0.20 |
| 4 | 28.93 | C23H28O11 | 480.152 | P | **Albiflorin** | **Monoterpene glucoside** | 3.6 | 1.62 |
| 5 | 30.02 | C23H28O11 | 480.200 | P | **Paeoniflorin** | **Monoterpene glucoside** | 14 | 3.47 |
| 6 | 33.5 | C21H22O9 | 418.100 | G | **Liquiritin (256.0736 fragment)** | **Flavonone O-glycoside** |  | 7.09 |
| 7 | 34.11 | C26H30O13 | 550.200 | G | Liquiritigenin-4'-apiosiyl-glucoside (256.0632 fragment) | Flavonone O-glycoside |  | 5.06 |
| 8 | 34.27 | C26H28O13 | 548.137 | S |  |  |  | 14.24 |
| 9 | 34.9 | C26H28O13 | 548.141 | S |  |  |  | 1.56 |
| 10 | 35.17 | C29H36O15 | 624.300 | S | Acteoside | Glycoside |  | 0.94 |
| 11 | 35.56 | C26H28O13 | 548.135 | S |  |  |  | 9.52 |
| 12 | 35.85 | C21H18O12 | 462.064 | S | **Scutellarin** | **Flavone O-glycoside** | 8 | 2.17 |
| 13 | 36.44 | C26H28O13 | 548.139 | S |  |  |  | 3.08 |
| 14 | 36.96 | C23H28O11 | 480.153 | P |  |  |  | 1.26 |
| 15 | 37.76 | C28H30O16 | 460.092 | S |  |  |  | 0.56 |
| 16 | 38.75 | C17H14O8 | 346.062 | S | Viscidulin III | Flavone |  | 1.92 |
| 17 | 39.86 | C22H20O12 | 476.081 | S |  |  |  | 2.62 |
| 18 | 40.14 | C22H22O9 | 430.114 | G | **Ononin** | **Isoflavonoid O-glycoside** | 3 | 1.30 |
| 19 | 40.36 | C15H12O4 | 256.064 | G | **Liquiritigenin** | **Flavanone** | 0.6 | 1.14 |
| 20 | 41.02 | C21H18O11 | 446.056 | S | **Baicalin** | **Flavone O-glycoside** | 48.8 | 100.00 |
| 21 | 41.02 | C21H18O12 | 462.050 | S |  |  |  | 1.58 |
| 22 | 42.91 | C21H18O11 | 446.072 | S |  |  |  | 14.15 |
| 23 | 43.82 | C21H18O11 | 446.076 | S |  |  |  | 4.59 |
| 24 | 43.82 | C22H20O12 | 476.082 | S |  |  |  | 6.02 |
| 25 | 44.25 | C21H18O10 | 430.080 | S |  |  |  | 8.43 |
| 26 | 44.63 | C22H20O11 | 460.083 | S | Oroxylin-7-O-Glucuronide | Flavone O-glycoside | 26 | 40.59 |
| 27 | 45.81 | C22H20O11 | 460.076 | S | Wogonoside | Flavone O-glycoside | 39.4 | 82.30 |
| 28 | 46.67 | C23H22O12 | 490.099 | S |  |  |  | 1.84 |
| 29 | 47.58 | C15H10O5 | 270.045 | S | **Apigenin** | **Flavone** | 0.8 | 0.86 |
| 30 | 48.76 | C16H12O6 | 300.051 | S | trihydoxymethoxyflavone | Flavone |  | 1.82 |
| 31 | 49.28 | C15H10O5 | 270.040 | S | **Baicalein** | **Flavone** | 14.0 | 14.62 |
| 32 | 52.12 | C48H72O21 | 984.451 | G | Licorce Saponin A3 | Glucuronide saponin |  | 2.13 |
| 33 | 53.57 | C44H64O18 | 880.408 | G | Acetylated Licorice Saponin G2 | Glucuronide saponin |  | 3.42 |
| 34 | 55.22 | C42H61O16 | 821.382 | G | Glyeurysaponin | Glucuronide saponin |  | 1.23 |
| 35 | 56.1 | C16H12O5 | 284.058 | S | **Wogonin** | **Flavone** | 5.7 | 11.44 |
| 36 | 56.65 | C42H62O17 | 838.374 | G | Licorice Saponin G2 | Glucuronide saponin |  | 4.79 |
| 37 | 57.41 | C19H18O8 | 374.093 | S | dihydroxytetramethoxyflavone | Flavone |  | 3.95 |
| 38 | 58.12 | C16H12O5 | 284.060 | S | Oroxylin A | Flavone | 2.4 | 3.82 |
| 39 | 59.67 | C42H62O16 | 822.405 | G | **Glycyrrhizin** | **Triterpenoid saponin glycoside** | 14.2 | 34.18 |

Thirty-nine peaks observed in extracts of PHY906 and HQT samples are listed with LC retention time, experimental mass, elemental formula, herbal source, chemical identification, chemical class, relative abundance and absolute quantity based on standard marker compounds. The extracted ion count peak intensities represented greater than 85% of the total ion current. The largest peak was Baicalin at 41.02 minute retention time and mass 446.056. The other 38 peaks ranged from 0.2% to 82.3% of this highest intensity peak. Of the 39 peaks, 25 were from (S), 10 were from (G), 3 were from (P) and 1 was from (Z) as confirmed by examination of the individual herbal ingredients and various ratios of the herbal ingredients. There was no overlap between the peaks from the four individual herbal ingredients. Ten of the 39 peaks were confirmed by standard marker compounds (bold). Thirteen additional peaks were assigned by comparisons with known compounds from each herbal ingredient using exact mass and high resolution MS/MS fragmentation patterns.
